# Incidence and severity of cytomegalovirus infection in seropositive heart transplant recipients

**DOI:** 10.1111/ctr.14982

**Published:** 2023-03-29

**Authors:** Bradley J. Gardiner, Jessica P. Bailey, Mia A. Percival, Beth A. Morgan, Victoria M. Warner, Sue J. Lee, C. Orla Morrissey, David M. Kaye, Anton Y. Peleg, Andrew J. Taylor

**Affiliations:** ^1^ Department of Infectious Diseases Alfred Health and Central Clinical School Monash University Melbourne Victoria Australia; ^2^ Pharmacy Department Alfred Health Melbourne Victoria Australia; ^3^ Department of Cardiology Alfred Health Melbourne Victoria Australia; ^4^ Department of Medicine Monash University Melbourne Australia; ^5^ Baker Heart & Diabetes Institute Melbourne Australia; ^6^ Department of Microbiology Biomedicine Discovery Institute Monash University Clayton Victoria Australia

**Keywords:** cytomegalovirus, heart transplant, recipient positive

## Abstract

**Background:**

The frequency and significance of cytomegalovirus (CMV) infection in seropositive (R+) heart transplant recipients (HTR) is unclear, with preventative recommendations mostly extrapolated from other groups. We evaluated the incidence and severity of CMV infection in R+ HTR, to identify risk factors and describe outcomes.

**Methods:**

R+ HTR from 2010 to 2019 were included. Antiviral prophylaxis was not routinely used, with clinically guided monitoring the local standard of care. The primary outcome was CMV infection within one‐year post‐transplant; secondary outcomes included other herpesvirus infections and mortality.

**Results:**

CMV infection occurred in 27/155 (17%) R+ HTR. Patients with CMV had a longer hospitalization (27 vs. 20 days, unadjusted HR 1.02, 95% CI 1.00–1.02, *p* = .01), higher rate of intensive care readmission (26% vs. 9%, unadjusted HR 3.46, 1.46–8.20, *p* = .005), and increased mortality (33% vs. 8%, unadjusted HR 10.60, 4.52–24.88, *p* < .001). The association between CMV and death persisted after adjusting for multiple confounders (HR 24.19, 95% CI 7.47–78.30, *p* < .001). Valganciclovir prophylaxis was used in 35/155 (23%) and was protective against CMV (infection rate 4% vs. 27%, adjusted HR .07, .01–.72, *p* = .025), even though those receiving it were more likely to have received thymoglobulin (adjusted OR 10.5, 95% CI 2.01–55.0, *p* = .005).

**Conclusions:**

CMV infection is common in R+ HTR and is associated with a high burden of disease and increased mortality. Patients who received valganciclovir prophylaxis were less likely to develop CMV infection, despite being at higher risk. These findings support the routine use of antiviral prophylaxis following heart transplantation in all CMV R+ patients.

## 1 BACKGROUND

Cytomegalovirus (CMV) remains a major contributor to morbidity and mortality in heart transplant recipients (HTR).^1^, ^2^ It causes direct clinical illness and indirectly contributes to poor outcomes including cardiac allograft vasculopathy.^3^, ^4^, ^5^, ^6^, ^7^ Preventative approaches are effective and include antiviral prophylaxis or pre‐emptive therapy.^8^, ^9^ Primary prophylaxis with valganciclovir has emerged as the dominant CMV preventative strategy and is associated with less CMV infection and improved outcomes.^10^, ^11^, ^12^, ^13^ Despite this, CMV infection remains a problem and is difficult to predict.^14^, ^15^, ^16^ The pre‐emptive approach can be challenging and resource‐intensive to implement. Routine use of prophylaxis increases the risk of side effects such as neutropenia, drug costs, and antiviral resistance.

Patients who acquire CMV from their transplant donor (donor positive/recipient negative, D+/R‐) are at the highest risk of infection and generally receive 6 months of valganciclovir prophylaxis.^17^, ^18^, ^19^, ^20^ Recipients who have had an infection with CMV prior to transplantation (R+ HTR) are considered intermediate risk unless they receive antilymphocyte agents such as thymoglobulin for induction or rejection.^21^, ^22^, ^23^ Their risk of CMV is less clear, with estimates in prior studies ranging widely.^24^, ^25^, ^26^, ^27^, ^28^, ^29^ Practice varies in these patients with many receiving 3‐6 months of prophylaxis but others undergoing virologic monitoring. Few dedicated studies have been performed in this subgroup, with recommendations mostly extrapolated from other solid organ transplant groups.^30^, ^31^

Given the widespread use of universal valganciclovir prophylaxis in many transplant programs, defining the burden of CMV infection in a contemporary cohort of R+ HTR who have not received prophylaxis is difficult. At our center, antiviral prophylaxis has been implemented selectively among R+ HTR who are considered at higher risk. The aims of this retrospective cohort study were to describe the frequency and severity of CMV infection in our R+ HTR, to understand the relationship between CMV and clinical outcomes, and to explore risk factors for CMV infection.

## 2 METHODS

### 2.1 Study design and population

Patients who received a heart transplant at our institution between 2010 and 2019 who had positive CMV serology prior to transplantation were eligible for inclusion. Patients were excluded if they died during or less than 72 h following the transplant surgery. Clinical data were obtained from hospital medical records. The study was approved by the Alfred Health Ethics Committee, with informed consent not required given its retrospective nature and minimal risk.

### 2.2 Immunosuppression and rejection protocols

Standard maintenance immunosuppression was with a calcineurin inhibitor (tacrolimus or cyclosporine), an antiproliferative drug (typically mycophenolate) and prednisolone. Basiliximab was used as a calcineurin inhibitor sparing agent for induction in patients at high risk for renal dysfunction. Acute cellular rejection was diagnosed on cardiac biopsy specimens according to the International Society for Heart and Lung Transplantation pathological scoring system.^32^ Biopsies were performed according to a standardized protocol, weekly for 6 weeks, fortnightly for 6 weeks, monthly for 3 months, then every 2 months for a further 6 months. Patients had additional biopsies if clinically indicated. Rejection grade 2 or higher was treated with intravenous methylprednisolone 500–1000 mg daily for 3 days. The rejection that did not respond to high‐dose steroids was treated with an anti‐lymphocyte agent (generally anti‐thymocyte globulin for cellular rejection and rituximab/plasmapheresis for antibody‐mediated rejection).

### 2.3 Outcomes

Our primary outcome, CMV infection, was defined as a positive test for CMV at any anatomical site (mostly serum viral load testing). Asymptomatic patients with an isolated low‐level positive test that spontaneously resolved without antiviral therapy who did not have any other clinical evidence for disease were not classified as CMV infection. Secondary outcomes included herpes simplex virus (HSV) and varicella zoster virus (VZV) infections, which were classified as localized if restricted to one anatomic site (e.g., single dermatomal shingles or oral HSV) or disseminated if beyond this. We also examined overall mortality.

### 2.4 CMV prophylaxis, diagnosis, and treatment

Antiviral prophylaxis was not routine in this cohort but rather targeted to patients considered high‐risk by their treating clinicians, such as those receiving an anti‐lymphocyte agent for induction or rejection. When used, patients received oral valganciclovir 450 mg twice daily for 3 months with doses adjusted for renal impairment. CMV viral load testing was performed on plasma using the Cobas AmpliPrep/TaqMan CMV assay (Roche Diagnostics, Mannheim, Germany) which has a detection range of 150–1 000 000 copies/mL (137–910 000 IU/mL, conversion factor .91). Viral load testing was performed when clinically indicated without routine asymptomatic surveillance. CMV end‐organ disease required laboratory confirmation of CMV plus clinical evidence of organ dysfunction, categorized as *proven* (positive pathology or PCR at a non‐blood site with attributable symptoms), *probable* (DNAemia plus attributable symptoms), or *possible* (DNAemia with clinical symptoms suggestive of end‐organ involvement but a potential alternative diagnosis present).^33^ CMV pneumonitis required evidence of a pulmonary infiltrate on imaging plus identification of CMV on lung biopsy (proven) or in BAL fluid (probable). CMV syndrome was defined as CMV DNAemia plus ≥2 of: fever for ≥2 days, new or increased malaise, neutropenia (<1.5 × 103/μL) or thrombocytopenia (<100 × 103/μL), elevated AST or ALT (>2× upper limit of normal). Standard treatment of CMV infection was with intravenous ganciclovir 5 mg/kg twice daily or oral valganciclovir 900 mg twice daily, adjusted for renal impairment. Duration of therapy was individualized and determined by clinical and virologic endpoints but was generally at least 2–3 weeks.

### 2.5 Statistical analysis

Categorical data were reported as counts with percentages, continuous data as means ± standard deviations if normally distributed and medians with interquartile ranges otherwise. Groups were compared using the Chi‐squared/Fishers’ exact test for categorical variables, Student's *T*‐test for normally distributed variables and the Wilcoxon rank sum test for non‐normally distributed continuous variables. Risk was quantified with odds ratios (OR) with 95% confidence intervals (CI), calculated using logistic regression. Kaplan‐Meier survival curves representing time to CMV infection were compared using the log‐rank test. Hazard ratios (HR) were calculated using Cox proportional hazards models and the proportional hazards assumption was confirmed by assessing Schoenfeld residuals and visualizing log‐log plots. There were two separate Cox analyses performed as part of this study. In the first analysis, CMV infection was the outcome and if patients died before 1 year without CMV they were censored as no event. Valganciclovir prophylaxis use was included as a time‐varying covariate. In the second, death was the outcome and CMV infection was the key covariate of interest (time‐varying). Censoring otherwise occurred at the earliest of loss to follow‐up or 1‐year post‐transplant. Patients who were diagnosed with CMV on autopsy were classified as having had CMV disease 1 day prior to death. Multivariable models were developed using a combined approach of a priori variable selection based on clinical reasoning as well as statistical factors including collinearity assessment and nested model comparisons using Akaike and Bayesian Information Criterion (AIC & BIC). *p*‐values of <.05 were considered statistically significant. All analyses were performed with Stata IC14.2.

## 3 RESULTS

### 3.1 Cohort description

**FIGURE 1:**
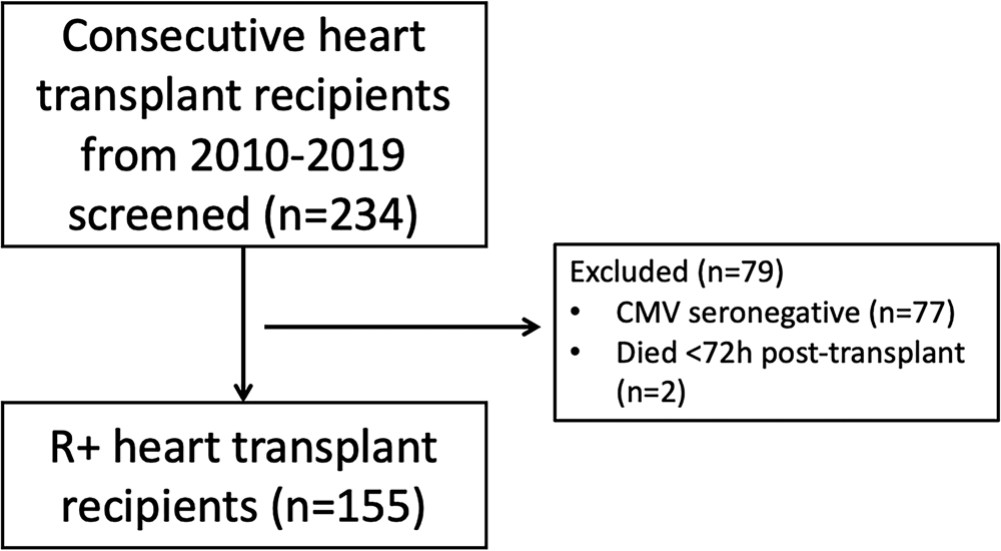
Number and flow of study participants.

A total of 155 R+ HTR were included (Figure 1). Median age was 53 years, 111 (72%) were male, 105 (68%) received basiliximab induction and 86 (56%) had CMV seropositive donors (Table 1). Thirteen (8%) received thymoglobulin induction. Valganciclovir prophylaxis was used in 35 (23%) patients for a median of 3.4 months (IQR 2.6–7.4 months). Three patients (2%) received prophylaxis with low‐dose valaciclovir for HSV/VZV. Rejection grade 2 or higher occurred in 108 (70%) patients, of whom 103 (66%) received high‐dose steroids and 26 (17%) received an antilymphocyte agent. Nineteen patients (12%) died, at a median of 2.9 months (IQR 1.1–4.5 months) post‐transplant. Six patients (4%) had an incomplete follow‐up, mostly because their care was transferred to another center.

**TABLE 1:** Baseline characteristics of the cohort of CMV recipient positive heart transplant recipients (*n* = 155).

**Characteristic**	**Details**
Age at transplant, median, IQR	53, 42–60
Male sex, no. (%)	111 (72%)
Calendar year of transplant, median, IQR	2015 2012–2018
Body mass index, mean ± SD	25.7 ± 3.8
Underlying cardiac disease, no. (%)	
Non‐ischemic cardiomyopathy	95 (61%)
Ischemic cardiomyopathy	43 (28%)
Congenital heart disease	7 (5%)
Other	10 (7%)
Pre‐transplant diabetes, no. (%)	21 (14%)
Previous transplant, no. (%)	9 (6%)
Pre‐transplant ventricular assist device, no. (%)	76 (49%)
Ischemic time (minutes), median, IQR	183, 155–250
CMV serostatus, no. (%)	
D+/R+	86 (56%)
D‐/R+	69 (45%)
EBV serostatus, no. (%) (*n* = 150)	
D+/R+	137 (91%)
D+/R‐	3 (2%)
D‐/R+	10 (7%)
Cardiac bypass time (minutes), median, IQR (*n* = 152)	142, 117–191
>1 organ transplanted, no. (%)	4 (3%)
Transplant related return to surgery, no. (%)	43 (28%)
Post‐operative ECMO, no. (%)	35 (23%)
Days intubated post‐transplant, median, IQR	3, 1–6
Days in ICU post‐transplant, median, IQR	8, 5–11
Readmission to ICU during index admission, no. (%)	18 (12%)
Duration of index admission (days), median, IQR	21, 15–30
Induction immunosuppression, no. (%)	
Basiliximab	105 (68%)
Mycophenolate	153 (99%)
Tacrolimus	58 (37%)
Thymoglobulin	13 (8%)
Immunosuppression on discharge post‐transplant (*n* = 144)	
Tacrolimus, no. (%)	74 (51%)
Mycophenolate, no. (%)	138 (96%)
Prednisolone, no. (%)	140 (97%)
Prednsiolone dose (mg), median, IQR	20 (20,20)
Antiviral prophylaxis	
Valganciclovir, no. (%)	35 (23%)
Valganciclovir duration, months, median, IQR	3.4, 2.6–7.4
Valaciclovir, no. (%)	3 (2%)
Valaciclovir duration, months, median, IQR	.5, .2–12
Rejection*, no. (%)	
High‐dose steroids	103 (66%)
Antilymphocyte agent	26 (17%)
Any antilymphocyte agent for induction or rejection, no. (%)	39 (25%)
Death within 1 year of transplant, no. (%)	19 (12%)
Months from transplant to death, median, IQR	2.9, 1.1–4.5
Loss to follow‐up <1 year, no. (%)	6 (4%)

Abbreviations: CMV, cytomegalovirus; D, donor; EBV, Epstein‐Barr virus; ECMO, extracorporeal membrane oxygenation; ICU, intensive care unit; IQR, interquartile range; IV, intravenous; R, recipient.

* Including all rejection episodes during the study period.

### 3.2 CMV infection

CMV infection occurred in 27/155 (17%) patients, at a median of 2.2 months (IQR 1.4–4.1 months) post‐transplant, with 17/27 (63%) and 23/27 (85%) of cases occurring before 3 and 6 months, respectively. This included 22/27 (82%) with end‐organ disease (Table 2), most commonly in the gastrointestinal tract (14/27, 52%). Four patients had CMV syndrome and one asymptomatic viremia. Six patients developed CMV reactivation during their index admission, but most patients with CMV (18/27, 67%) became unwell after discharge and required readmission, for a median of 11 days (IQR 5–23 days). The median antiviral treatment duration was 5.1 weeks, with 14/27 (52%) receiving intravenous ganciclovir at least initially. VZV infection occurred in nine (6%) patients (four localized, five disseminated) and HSV in 25 (16%), five of whom had disseminated infection. All of these cases were confirmed by PCR except for two cases of localized oral HSV infection which were clinically diagnosed and responded to antiviral treatment.

**TABLE 2:** Cytomegalovirus and other herpesvirus infection clinical outcome details (*n* = 155).

**Characteristic**	**Details**
CMV infection, no. (%)	27 (17%)
Asymptomatic viremia	1 (4%)
CMV syndrome	4 (15%)
End‐organ disease	22 (82%)
Site of end‐organ disease, no. (%)	
Gastrointestinal	14 (52%)
Pulmonary	3 (11%)
Disseminated	3 (11%)
Other	3 (11%)
Type of end‐organ disease, no. (%)	
Proven	8 (36%)
Probable	5 (23%)
Possible	9 (41%)
Peak viral load (IU/mL), median, IQR (*n* = 20)	1250, 221–6539
Days of symptoms prior to CMV onset, median, IQR (*n* = 26)	9, 2–15
Months from transplant to CMV diagnosis, median, IQR	2.2, 1.4–4.1
Admitted for CMV, no. (%)	
Readmitted	18 (67%)
Occurred during post‐transplant admission	6 (22%)
Outpatient only	3 (11%)
Length of stay for readmitted patients (days), median, IQR	11, 5–23
Antiviral treatment type, no. (%)	
Oral valganciclovir	6 (22%)
IV ganciclovir	7 (26%)
IV ganciclovir & oral valganciclovir	7 (26%)
None	7 (26%)
Antiviral treatment duration (weeks), median, IQR	5.1, 2.3–7.6
VZV infection, no. (%)	9 (6%)
Localised	4 (44%)
Disseminated	5 (56%)
Onset (days), median, IQR	45, 26–91
HSV infection, no. (%)	25 (16%)
Oral	15 (60%)
Disseminated	5 (20%)
Other	5 (20%)
Onset (days), median, IQR	24, 18–47

Abbreviations: CMV, cytomegalovirus; D, donor; HSV, herpes simplex virus; IV, intravenous; R, recipient; VZV, varicella zoster virus.

**FIGURE 2:**
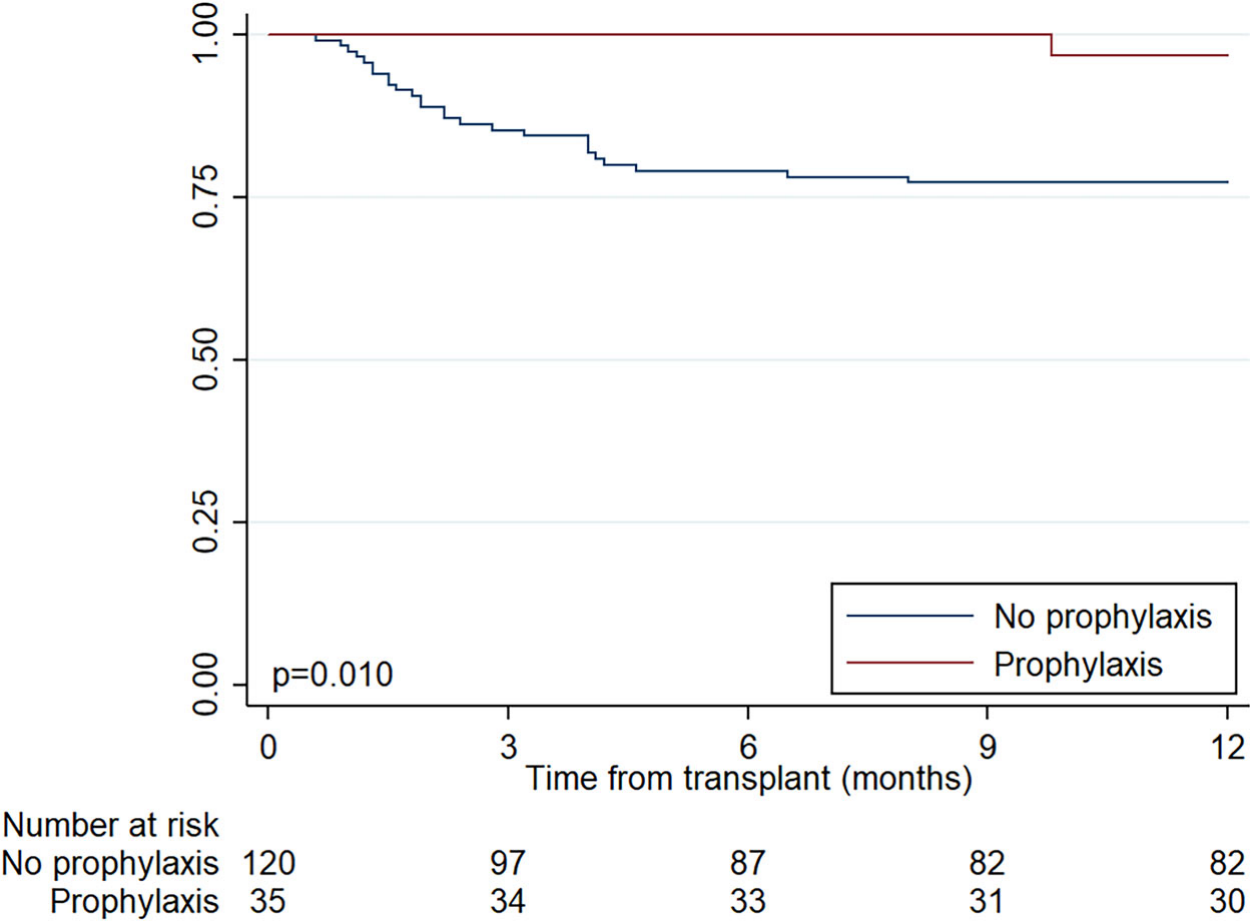
Unadjusted Kaplan‐Meier curves of time to cytomegalovirus infection, by use of valganciclovir prophylaxis (*n* = 155). *p*‐value refers to log‐rank test results.

Baseline characteristics stratified by CMV infection status are shown in Table 3. Patients with CMV infection were more likely to have received post‐transplant extra‐corporeal membrane oxygenation (ECMO), were readmitted to the ICU more often and had a longer duration of hospitalization. Those who received valganciclovir prophylaxis were much less likely to develop CMV infection (4% vs. 27%, unadjusted HR .13, 95% CI .017–.92, *p* = .042, Figure 2). After adjusting for ICU readmission, length of stay, and use of thymoglobulin, valganciclovir prophylaxis use remained highly protective against CMV (adjusted HR .07, 95% CI .01–.72, *p* = .025).

**TABLE 3:** Characteristics of patients with CMV infection compared to those without, with unadjusted and adjusted hazard ratios for CMV (*n* = 155).

**Description**	**No CMV infection (*n* = 128)**	**CMV infection (*n* = 27)**	**Unadjusted HR, 95% CI**	***p*‐value**	**Adjusted HR, 95% CI**	***p*‐value**
Age at transplant, median, IQR	53, 42–60	55, 43–61	1.01, .98–1.04	.52		
Male sex, no. (%)	94 (73%)	17 (63%)	.64, .29–1.40	.27		
Calendar year of transplant, median, IQR	2015, 2012–2018	2015, 2012–2017	.99, .87–1.13	.89		
Body mass index, mean ± SD	25.5 ± 3.8	26.5 ± 3.5	1.07, .96–1.19	.21		
Pre‐transplant diabetes, no. (%)	19 (15%)	2 (7%)	.54, .13–2.27	.40		
Previous transplant, no. (%)	6 (5%)	3 (11%)	2.46, .74–8.17	.14		
Recipient EBV seropositive, no. (%)	126 (98%)	26 (96%)	.42, .057–3.09	.39		
Pre‐transplant ventricular assist device, no. (%)	63 (49%)	13 (48%)	.92, .43–1.97	.84		
Ischemic time (minutes), median, IQR	183, 155–238	211, 147–254	1.00, 1.00–1.01	.98		
Donor CMV seropositive, no. (%)	69 (54%)	17 (63%)	1.48, .68–3.23	.33		
Cardiac bypass time (mins), median, IQR (*n* = 152)	143, 118–192	138, 115–170	1.00, .99–1.00	.42		
>1 organ transplanted, no. (%)	3 (2%)	1 (4%)	2.17, .29–16.04	.45		
Post‐operative ECMO, no. (%)	26 (20%)	9 (33%)	2.27, 1.02–5.06	.045		
Days intubated post‐transplant, median, IQR	3, 1–6	4, 1–6	1.02, .96–1.09	.51		
Days in ICU post‐transplant, median, IQR	7, 5–11	8, 5–13	1.02, .99–1.05	.14		
Readmission to ICU during index admission, no. (%)	11 (9%)	7 (26%)	3.46, 1.46–8.20	.005	2.13, .61–7.47	.24
Duration of index admission (days), median, IQR	20, 15.5–28	27, 15–43	1.01, 1.00–1.02	.011	1.02, 1.00–1.03	.033
Induction immunosuppression, no. (%)						
Basiliximab	88 (69%)	17 (63%)	.81, .37–1.78	.61		
Thymoglobulin	12 (9%)	1 (4%)	.40, .05–2.92	.36		
Valganciclovir prophylaxis, no. (%)^a^	34 (27%)	1 (4%)	.13, .017–.92	.042	.07, .01–.72	.025
Valaciclovir prophylaxis, no. (%)	2 (2%)	1 (4%)	2.16, .29–16.0	.45		
Rejection, no. (%)^b^						
High‐dose steroids for rejection	85 (84%)	16 (59%)	.71, .33–1.52	.38		
Antilymphocyte agent for rejection	22 (17%)	3 (11%)	.61, .18–2.02	.42		
Any antilymphocyte agent for induction or rejection, no. (%)	34 (27%)	4 (15%)	.50 (.17–1.44)	.20	.69 (.22–2.20)	.53
Death within 1 year of transplant, no. (%)	10 (8%)	9 (33%)	10.60, 4.52–24.88	<.001		

Abbreviations: CMV, cytomegalovirus; EBV, Epstein‐Barr virus; ICU, intensive care unit; IQR, interquartile range.

^a^ Analyzed as time varying.

^b^ Includes only rejection episodes that occurred prior to CMV infection.

### 3.3 Valganciclovir prophylaxis use

Because of the targeted use of prophylaxis in our cohort and the known association between this and the timing and likelihood of CMV infection, we wanted to understand the differences between the 35 (23%) patients who did and the 120 (77%) who did not receive prophylaxis (Table S1). Patients who received prophylaxis were transplanted more recently, had a longer cardiac bypass time, spent more days in the ICU post‐transplant, had a longer overall hospital length of stay, and were significantly more likely to have received thymoglobulin induction (31% vs. 2%, unadjusted OR 27.04, 95% CI 5.63–129.88, *p* < .0001). After multivariate analysis, the only use of thymoglobulin (adjusted OR 10.5, 95% CI 2.01–55.0, *p* = .005) and calendar year (adjusted OR 1.32 per year, 95% CI 1.10–1.57, *p* = .003) remained independently associated with valganciclovir prophylaxis use.

### 3.4 Mortality

Of the 19 patients who died within the first post‐transplant year, 9 (47%) had CMV infection and two had disseminated VZV/HSV, including one patient who had reactivation of all three herpesviruses. Two cases of disseminated CMV infection were not diagnosed until post‐mortem examination. Overall, 10/155 (6.5%) patients died in the setting of a significant herpesvirus infection. Many of these deaths were clearly related to the infection, in other cases the contribution was uncertain (Table S2).

**FIGURE 3:**
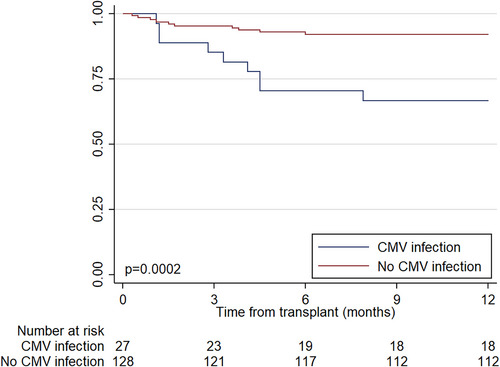
Unadjusted Kaplan‐Meier curves of time to death, by cytomegalovirus infection status (*n* = 155). *p*‐value refers to log‐rank test results.

Several factors were identified as being associated with increased mortality (Table 4). Many of these were correlated with each other and reflected increased severity of illness, such as post‐operative ECMO, extended intubation/ICU length of stay, and ICU readmission. Others were indicative of multiple comorbidities, such as a prior transplant or receipt of a second organ. Patients who developed CMV infection had a much higher mortality than those who did not (47% vs. 13%, HR 18.19, 95% CI 6.46–51.30, *p* < .001, Figure 3). This association persisted after adjusting for multiple key confounders (adjusted HR 24.19, 95% CI 7.47–78.30, *p* < .001). There was a trend to lower mortality in patients who received prophylaxis (2/35, 6% vs. 17/120, 15%) but this did not reach statistical significance (unadjusted HR 0.69, 95% CI 0.20–2.37, *p* = .55, Figure S1).

**TABLE 4:** Characteristics of patients who died within 1 year post‐transplant compared to those who survived, with unadjusted and adjusted hazard ratios for death (*n* = 155).

**Description**	**Survived** **(*n* = 136)**	**Died** **(*n* = 19)**	**Unadjusted HR, 95% CI**	***p*‐value**	**Adjusted HR, 95% CI**	***p*‐value**
Age at transplant, median, IQR	53, 42–60	54, 41–65	1.02,.98–1.06	.39		
Male sex, no. (%)	100 (74%)	11 (58%)	.52, .21–1.29	.16		
Calendar year of transplant, median, range	2015, 2013–2017	2015, 2012–2018	1.01, .86–1.17	.93		
Body mass index, mean ± SD	25.7±3.7	25.2±4.2	.96, .86–1.09	.55		
Pre‐transplant diabetes, no. (%)	17 (13%)	4 (21%)	1.81, .60–5.46	.29		
Previous transplant, no. (%)	6 (4%)	3 (14%)	3.65, 1.06–12.55	.040	3.51, .91–13.54	.068
Recipient EBV seropositive, no. (%)	134 (99%)	18 (95%)	.38, .51–2.85	.35		
Pre‐transplant ventricular assist device, no. (%)	69 (51%)	7 (37%)	.59, .23–1.49	.26		
Ischemic time (minutes), median, IQR	179, 150–240	212, 181–280	1.00, 1.00–1.01	.21		
Donor CMV seropositive, no. (%)	74 (54%)	12 (63%)	1.39, .55–3.53	.49		
Cardiac bypass time (mins), median, IQR (*n* = 152)	139, 116–188	162, 140–247	1.01, 1.00–1.01	.031	1.01, 1.00–1.02	.001
>1 organ transplanted, no. (%)	2 (1%)	2 (11%)	6.12, 1.41–26.67	.016		
Post‐operative ECMO, no. (%)	21 (15%)	14 (74%)	12.15, 4.37–33.78	<.001		
Days intubated post‐transplant, median, IQR	3, 1–5	7, 3–14	1.09, 1.05–1.13	<.001		
Days in ICU post‐transplant, median, IQR	7, 5–10	20, 8–34	1.06, 1.04–1.08	<.001		
Readmission to ICU during index admission, no. (%)	10 (7%)	8 (42%)	6.22, 2.50–15.49	<.001	3.00, 1.13–7.96	.028
Duration of index admission (days), median, IQR	20, 15–27	37, 28–54	1.02, 1.01–1.03	<.001		
Induction immunosuppression, no. (%)						
Basiliximab	89 (65%)	16 (84%)	2.71, .79–9.31	.11		
Thymoglobulin	11 (8%)	2 (11%)	1.30, .30–5.63	.73		
Valganciclovir prophylaxis, no. (%)^a^	33 (24%)	2 (11%)	.69, .20–2.37	.55		
Valaciclovir prophylaxis, no. (%)	1 (.7%)	2 (11%)	7.06, 1.62–30.77	.009		
Rejection						
High‐dose steroids, no. (%)	92 (38%)	11 (58%)	.64, .26–1.59	.34		
Antilymphocyte agent, no. (%)	24 (18%)	2 (11%)	.57, .13–2.46	.45		
Any antilymphocyte agent for induction or rejection, no. (%)	35 (26%)	4 (21%)	.78 (.26–2.35)	.66		
CMV infection, no (%)^a^	18 (13%)	9 (47%)	18.19, 6.46–51.30	<.001	24.19, 7.47–78.30	<.001

Abbreviations: CMV, cytomegalovirus; EBV, Epstein‐Barr virus; ICU, intensive care unit; IQR, interquartile range.

^a^ Analyzed as time varying.

## 4 DISCUSSION

CMV remains an important opportunistic infection in HTR and contributes to poor outcomes, though the risk and significance in R+ patients has not been well defined. In this study we have described a large contemporary cohort of R+ HTR who mostly did not receive antiviral prophylaxis. CMV reactivation, which was mostly identified in the context of symptomatic clinical disease, occurred in one‐fifth of patients and was associated with worse post‐transplant outcomes including a significant burden of morbidity and prolonged hospitalization. Most importantly, we observed a significantly increased mortality risk among patients who experienced CMV infection. Patients who received valganciclovir prophylaxis were much less likely to develop CMV infection, despite being higher risk. CMV predominantly occurred within the first few months post‐transplant, often in patients hospitalized for extended periods experiencing other complications. This was more common, earlier, and more severe than we had expected. Several deaths that could have been related to CMV occurred early post‐transplant, including two patients with disseminated disease that was not diagnosed until post‐mortem examination.

Estimates of the frequency of CMV infection amongst R+ patients vary widely. Most prior studies combine serogroups, and those published since 2010 have almost all used routine prophylaxis for 3–6 months post‐transplant. For example, in one study which included 109 R+ HTR who received 3 months of prophylaxis, 13 (12%) developed CMV DNAemia within 1 year, but none developed CMV disease.^34^ Johansson et al. described 165 R+ HTR who did not receive prophylaxis, and 63/165 (38%) developed CMV infection including 32/165 (19%) with CMV disease, consistent with our findings.^3^ Similarly, Kocher et al. described 210 R+ HTR who received only CMV immunoglobulin prophylaxis, CMV infection occurred in 76/210 (36%), CMV disease in 41/210 (20%).^26^

Our study is one of the first to specifically evaluate the risk of CMV infection in a large contemporary cohort of R+ HTR, most of whom did not receive antiviral prophylaxis. Patients were described in detail and analyzed using robust statistical methodology that controlled for a variety of potential confounders. Our endpoint was robust with patients having clinically significant CMV infection, and we explored the significance of this in terms of overall mortality. However, there are some limitations which should be considered when interpreting our results. This was a single‐center, retrospective study with statistical power limited by sample size and the number of cases and outcomes, which meant we could not control for all potential confounders in our multivariable models. Variable selection was challenging, and we needed to balance including important confounders with the risk of overfitting. We performed several exploratory analyses prior to selecting our final models. Because all models considered were consistent with respect to the associations between prophylaxis use, CMV infection and death, the final models presented were clinically relevant, included non‐collinear confounders, did not violate the proportional hazards assumption, and provided good fit according to AIC and BIC when compared with other models. Testing for CMV reactivation was clinically driven so some cases of asymptomatic CMV viremia may have been missed, however the clinical significance of such cases is less clear. Understanding the contribution of CMV infection to death in complex cases is difficult, and autopsies were infrequently performed. Our cohort was compiled over a 10‐year period, and while patients who received prophylaxis tended to be transplanted more recently, there was no evidence of significant temporal differences that would have influenced our primary findings. Many of our CMV cases occurred early post‐transplant, making it difficult to explore the relationships between various post‐transplant factors and CMV. While our data collection was comprehensive, there is a possibility of residual confounding due to factors such as the level of immunosuppression, which is difficult to quantify but may be associated with CMV infection and/or death.

In conclusion, CMV infection is an important contributor to poor outcomes in R+ HTR. Our findings support the routine use of preventative strategies for CMV and other herpesviruses in all R+ HTR, either pre‐emptive therapy or routine antiviral prophylaxis, which has now been implemented at our center. It remains difficult to predict which individual patients will develop CMV, and further studies are required to explore individualized risk prediction enabling specific targeting of the patients at highest risk. Additional work is needed to understand the optimal duration of prophylaxis which may vary between patients and explore other strategies for reducing the burden of CMV in transplant recipients, improving long‐term outcomes.

## AUTHOR CONTRIBUTIONS

Bradley J. Gardiner was involved in all aspects of the study including design, data collection, analysis, results interpretation, and manuscript preparation. Andrew J. Taylor provided supervision, oversight, results interpretation, and manuscript review. Jessica P. Bailey, Beth A. Morgan, and Mia A. Percival contributed to data collection and manuscript writing. Mia A. Percival and Sue J. Lee performed the analysis. C. Orla Morrissey, David M. Kaye, and Anton Y. Peleg assisted with data interpretation and manuscript review. All authors have reviewed the manuscript and are in agreement with its content.

## CONFLICTS OF INTEREST STATEMENT

Bradley J. Gardiner has participated in advisory boards and received institutional research funding from Takeda. All other authors report no conflicts of interest.

## Supporting information

Supp Information

## Data Availability

The data that support the findings of this study are available from the corresponding author upon reasonable request.
