# Supplementary material for: Incidence and severity of cytomegalovirus infection in seropositive heart transplant recipients
Source: Clin Transplant. 2023 Mar 29;37(6):e14982. doi: 10.1111/ctr.14982 (PMC10909407; doi:10.1111/ctr.14982)
Supplement: Supplementary file 1 — Supp Information [file CTR-37-e14982-s001.docx]

**Incidence and severity of cytomegalovirus infection in seropositive heart transplant recipients**

Bradley J. Gardiner^1^, Jessica P. Bailey^2^, Mia A. Percival^2^, Beth A. Morgan^1^, Victoria M. Warner^2,3^, Sue J. Lee^1^, C. Orla Morrissey^1^, David M. Kaye^3, 4^, Anton Y. Peleg^1,5^, Andrew J. Taylor^3,4^

^1^Department of Infectious Diseases, Alfred Health and Central Clinical School, Monash University, Melbourne, Victoria, Australia

^2^Pharmacy Department, Alfred Health, Melbourne, Victoria, Australia

^3^Department of Cardiology, Alfred Health, Melbourne, Victoria, Australia

^4^Baker Heart & Diabetes Institute, Melbourne, Australia; Department of Medicine, Monash University, Melbourne, Australia

^5^Biomedicine Discovery Institute, Department of Microbiology, Monash University, Clayton, Victoria, Australia

*Supplementary Materials – Table of Contents*

1. Supplementary Figures
2. Supplementary Tables

**1. Supplementary Figures**

**Figure S1:** Unadjusted Kaplan-Meier curve of time to death by valganciclovir prophylaxis use (n=155).


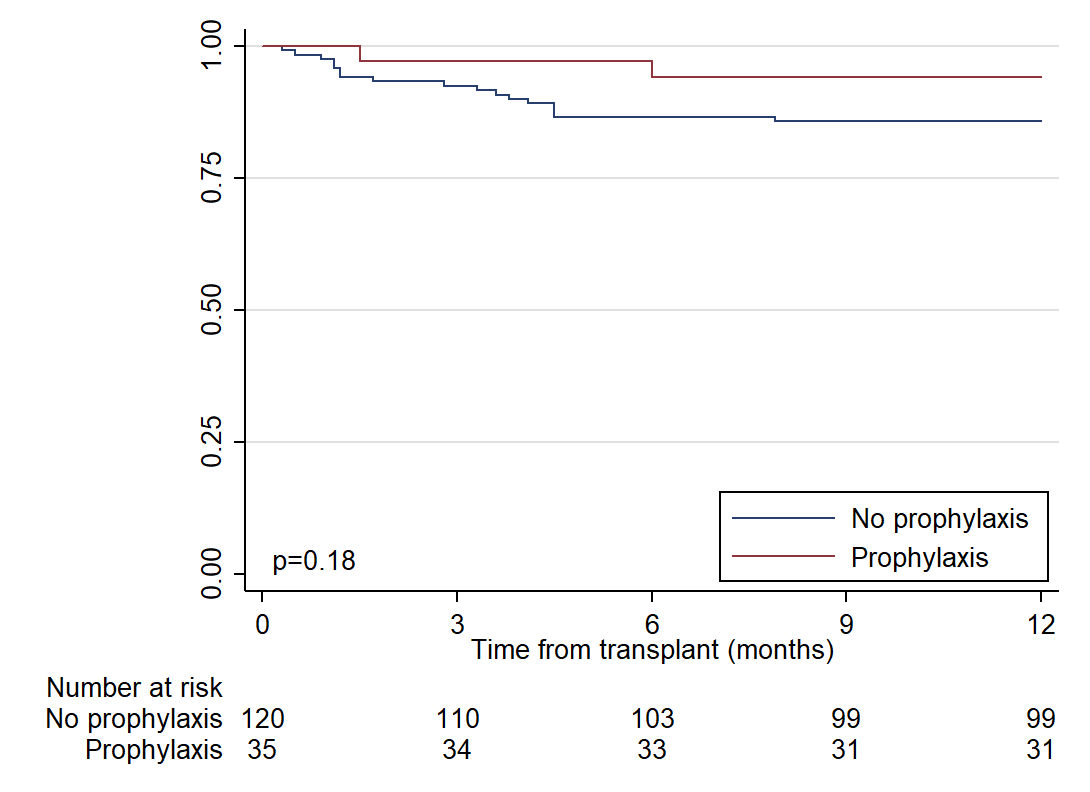


P-value refers to log-rank test result.

**2. Supplementary Tables**

**Table S1:** Characteristics of patients who received valganciclovir prophylaxis compared to those who did not, with unadjusted and adjusted odds ratios for prophylaxis use (n=155).

| **Description** | **No prophylaxis (n=120)** | **Prophylaxis**  **(n=35)** | **Unadjusted OR, 95% CI** | **p-value** | **Adjusted OR, 95% CI** | **p-value** |
| --- | --- | --- | --- | --- | --- | --- |
| Age at transplant, median, IQR | 52, 41.3-59.7 | 54.1, 47.1-61.8 | 1.02, 0.98-1.05 | 0.32 |  |  |
| Male sex, no. (%) | 84 (70%) | 27 (77%) | 1.45, 0.60-3.49 | 0.41 |  |  |
| Calendar year of transplant, median, IQR | 2014, 2012-2016 | 2018, 2017-2018 | 1.43, 1.21-1.70 | <0.0001 | 1.32, 1.10-1.57 | 0.003 |
| Body mass index, mean ± SD | 25.7 ± 4.0 | 25.7 ± 3.0 | 1.00, 0.91-1.11 | 0.97 |  |  |
| Pre-transplant diabetes, no. (%) | 18 (15%) | 3 (9%) | 0.53, 0.15-1.92 | 0.34 |  |  |
| Previous transplant, no. (%) | 5 (4%) | 4 (11%) | 2.97, 0.75-11.72 | 0.12 |  |  |
| Recipient EBV seropositive, no. (%) | 118 (98%) | 34 (97%) | 0.58, 0.051-6.55 | 0.66 |  |  |
| Pre-transplant ventricular assist device, no. (%) | 63 (53%) | 13 (37%) | 0.53, 0.25-1.16 | 0.11 |  |  |
| Ischemic time (minutes), median, IQR | 184, 153-240 | 182, 159-256 | 1.00, 1.00-1.01 | 0.46 |  |  |
| Donor CMV seropositive, no. (%) | 65 (54%) | 21 (60%) | 1.27, 0.59-2.73 | 0.54 |  |  |
| Cardiac bypass time (minutes), median, IQR (n=152) | 136, 115-185 | 175, 140-211 | 1.01, 1.00-1.01 | 0.016 | 1.01, 1.00-1.01 | 0.14 |
| >1 organ transplanted, no. (%) | 2 (2%) | 2 (6%) | 3.58, 0.49-26.36 | 0.21 |  |  |
| Post-operative ECMO, no. (%) | 26 (22%) | 9 (26%) | 1.25, 0.52-3.00 | 0.62 |  |  |
| Days intubated post-transplant, median, IQR | 3, 1-6 | 4, 2-7 | 1.06, 100-1.13 | 0.052 |  |  |
| Days in ICU post-transplant, median, IQR | 7, 5-10 | 9, 5-17 | 1.04, 1.00-1.07 | 0.034 |  |  |
| Readmission to ICU during index admission, no. (%) | 14 (12%) | 4 (11%) | 0.98, 0.30-3.18 | 0.97 |  |  |
| Duration of index admission (days), median, IQR | 20, 15-28 | 27, 17-33 | 1.02, 1.00-1.03 | 0.036 | 1.01, 0.99-1.03 | 0.32 |
| Induction immunosuppression, no. (%)    Basilixumab    Thymoglobulin | 79 (34%)  2 (2%) | 26 (74%)  11 (31%) | 1.50, 0.64-3.50  27.04, 5.63-129.88 | 0.35  <0.0001 | 10.5, 2.01-55.0 | 0.005 |
| Rejection  High-dose steroids, no. (%)  Antilymphocyte agent, no. (%) | 79 (66%)  15 (13%) | 24 (69%)  11 (31%) | 1.13, 0.51-2.54  3.21, 1.31-7.86 | 0.76  0.011 |  |  |
| Death, no. (%) | 17 (15%) | 2 (6%) | 0.65, 0.20-2.03 | 0.45 |  |  |

CMV, cytomegalovirus. EBV, Epstein-Barr virus. ECMO, extracorporeal membrane oxygenation. ICU, intensive care unit. IQR, interquartile range. IV

**Table S2:** Individual details of the patients who died following CMV, VZV or HSV infection (n=10).

| **Age/ sex** | **Underlying condition** | **Antiviral prophylaxis** | **Day of CMV diagnosis** | **Peak viral load (IU/mL)** | **Clinical details** | **Day of death** | **Autopsy findings** |
| --- | --- | --- | --- | --- | --- | --- | --- |
| 52/M | Ischemic CM | 3 weeks  valganciclovir | 19 | 1250 (blood) 25900 (BAL) | Extended ICU admission with sepsis, ischaemic foot, VRE bacteremia, CMV pneumonitis. Died following sudden cardiac arrest. | 37 | NP |
| 17/F | Postpartum CM | None | 124 | NP | Readmitted post-transplant with falls and abdominal pain. Developed worsening respiratory failure, deteriorated and died with multiorgan failure. | 124 | Disseminated CMV infection (lungs, liver, kidney, adrenals and stomach). Ischemic nephropathy with cortical infarcts, pulmonary infarcts. |
| 38/F | Postpartum CM | None | 40 | 3878 | CMV infection with neutropenia, thrombocytopenia, fevers, diarrhoea. Concurrent disseminated VZV & HSV infection with meningoencephalitis. Complications including post-operative bleeding causing cardiac tamponade requiring ECMO, AKI & sepsis. | 86 | NP |
| 59/F | Ischemic CM | 2 weeks valaciclovir | 68 | 1930 | Readmitted with nausea, vomiting, diarrhea and fevers. Underwent small bowel resection. Segments of small bowel showing acute ulceration, strongly positive for CMV. Associated *Pseudomonas* bacteraemia & *E. coli* UTI. Died from GI bleed following small bowel resection. | 99 | Disseminated angioinvasive aspergillosis (left parietal infarcts, renal infarct), DIC. No evidence of CMV infection. |
| 61/M | Dilated CM | None | 39* | CMV negative | Disseminated VZV & HSV (cutaneous, encephalitis) with multiple cortical infarcts, severe acute myelitis. | 51 | VZV meningoencephalitis with brainstem involvement. |
| 54/M | Ischemic CM | None | 55 | 1000000 | Readmitted with diarrhoea and sternal pain. Polymicrobial sepsis, sternal osteomyelitis (VRE, *Pseudomonas* UTI, ESBL Klebsiella groin collection). CMV colitis. | 136 | NP |
| 72/M | Nonischemic CM | None | 127 | +<150 (blood)  4171 (BAL) | Septic shock, pneumonia. Bilateral pneumothoracies. *Staph aureus* bacteremia. | 138 | NP |
| 66/M | Ischemic CM | None | 27 | 1312 | CMV syndrome with fevers, thrombocytopenia, neutropenia, hepatitis. Died with ischaemic bowel (no CMV on histology from bowel resection). | 32 | NP |
| 25/F | Chronic allograft vasculopathy (second transplant) | None | 38 | NP | Worsening nausea, vomiting, diarrhea day 8 post-transplant. Catastrophic oesophageal bleed not amenable to intervention. | 38 | Extensive GI CMV disease with esophageal ulceration, transmural necrosis & perforation. CMV enteritis proximal small bowel. |
| 67/M | Nonischemic CM | None | 32 | 34612 | Multiple complications including rejection, recurrent CMV infection, pulmonary mucormycosis, polymicrobial pneumonia & empyema. | 239 | NP |

AKI, acute kidney injury. BAL, bronchoalveolar lavage. CM, cardiomyopathy. CMV, cytomegalovirus. DIC, disseminated intravascular coagulation. ECMO, extracorporeal membrane oxygenation. ESBL, extended spectrum beta-lactamase. GI, gastrointestinal. ICU, intensive care unit. NP, not performed. UTI, urinary tract infection. HSV, herpes simplex virus. VRE, vancomycin-resistant Enterococcus. VZV, varicella zoster virus.

*VZV & HSV only, no CMV.
